# Testing the Significance of Ranked Gene Sets in Genome-wide Transcriptome **Profiling Data Using Weighted Rank Correlation Statistics**

**DOI:** 10.2174/0113892029280470240306044159

**Published:** 2024-03-11

**Authors:** Min Yao, Hao He, Binyu Wang, Xinmiao Huang, Sunli Zheng, Jianwu Wang, Xuejun Gao, Tinghua Huang

**Affiliations:** 1 College of Animal Science, Yangtze University, Jingzhou, Hubei, 434025, China;; 2 College of Agriculture, Yangtze University, Jingzhou, Hubei, 434025, China

**Keywords:** Flaver, ranked gene set, enrichment analysis, weighted rank correlation, GSEA, GOStats, transcription factor

## Abstract

**Background:**

Popular gene set enrichment analysis approaches assumed that genes in the gene set contributed to the statistics equally. However, the genes in the transcription factors (TFs) derived gene sets, or gene sets constructed by TF targets identified by the ChIP-Seq experiment, have a rank attribute, as each of these genes have been assigned with a p-value which indicates the true or false possibilities of the ownerships of the genes belong to the gene sets.

**Objectives:**

Ignoring the rank information during the enrichment analysis will lead to improper statistical inference. We address this issue by developing of new method to test the significance of ranked gene sets in genome-wide transcriptome profiling data.

**Methods:**

A method was proposed by first creating ranked gene sets and gene lists and then applying weighted Kendall's tau rank correlation statistics to the test. After introducing top-down weights to the genes in the gene set, a new software called “Flaver” was developed.

**Results:**

Theoretical properties of the proposed method were established, and its differences over the GSEA approach were demonstrated when analyzing the transcriptome profiling data across 55 human tissues and 176 human cell-lines. The results indicated that the TFs identified by our method have higher tendency to be differentially expressed across the tissues analyzed than its competitors. It significantly outperforms the well-known gene set enrichment analyzing tools, GOStats (9%) and GSEA (17%), in analyzing well-documented human RNA transcriptome datasets.

**Conclusions:**

The method is outstanding in detecting gene sets of which the gene ranks were correlated with the expression levels of the genes in the transcriptome data.

## 1 INTRODUCTION

Testing gene sets' significance in genome-wide transcriptome profiling data has been intensively studied recently. A typical computational issue in transcriptome profiling data analysis is deciding, given a biological process, to see if a predefined gene set has played roles in and thus related to this process. As we have summarized previously, the requirements for completing this task are: (i) establish the differential expression profiles of a particular biological process of interest; (ii) construct the predefined gene sets; (iii) discover the key gene sets statistically using data collected in requirements (i) and (ii). The RNA-Seq and other alternative techniques are increasingly affordable and can produce the differential expression gene list, which solved requirement (i) readily [1, 2].

Stat-of-the-art signaling pathway databases [3], GO annotation databases [4], computational TF targets prediction tools, and Chip-Seq techniques [5, 6] were available, providing multiple solutions for requirement (ii). The hypergeometric and Fisher's exact statistics-based enrichment analysis methods and their variants [7-10] and the Kolmogorov–Smirnov statistics-based enrichment analysis methods and their variants [11, 12] provided multiple options for the requirement (iii). The representative software implementing these methods are GOStats [13], ClusterProfiler [14], GSEA [12], WebGestalt [15], and others [11]. All these software require gene sets and a gene list as input sources. The inputs for GOStats are gene vectors for both gene sets and gene lists [7-10]. The input for ClusterProfiler and GSEA software, which both implemented the GSEA methods, is the gene sets, vectors of genes similar to GOStats, and a gene list with rank of expression values, which is different [12]. As we have pointed out earlier, most of the gene sets have an extra attribute, such as the putative TFs targets predicted by the Grit software [16] and other alternatives like FIMO [17] and Pscan [18], or targets identified by the ChIP-Seq experiment. Each of these targets have been assigned with a *p*-value which indicates the true or false possibilities of the TF binding sites. Ignoring this information during the enrichment analysis will lead to improper statistical inference. In this study, TF-derived gene sets with rank information were created and a description of our previously developed alternative gene set enrichment analysis software “Flaver” was presented. This tool addressed the question of finding the significant correlations between ranked gene sets and ranked gene lists by implementing the weighted Kendall's tau rank correlation statistics and adopting new ways of constructing the weighting functions. The tool performs differently than its competitors in analyzing ranked gene sets within the ranked gene list. Applications of Flaver in the human transcriptome data has yielded promising results, demonstrating its desirability as a gene set discovery tool.

## 2 MATERIALS AND METHODS

### 2.1 The Weighted Kendall's Tau Statistics

The method developed in this study tested the significance of the correlation between the rank orders for genes in the gene set and their corresponding rank orders within the gene list. It emphasizes items in the gene set based on top-down weighting functions. Items with higher weights will be emphasized, and those with lower weights will be de-emphasized. As illustrated by Shieh, the relationships between the gene set and the gene list, in terms of agreement in the ranks, in gene set enrichment analysis can be measured by a weighted rank correlation [19]. Let *S_i_* and *L_i_*, *i* = 1, …, *n* be the ranks of the gene set and gene list, respectively. Further, let (*i*, *R_i_*), *i* = 1, …, *n*, be paired ranks, where *R_i_* is a rank entity of *L* whose corresponding *S* has rank *i* among *S_j_*, *j* = 1, …, *n*. As discovered by Shieh, the weighted Kendall's tau has the form of Eq. 1 [19].


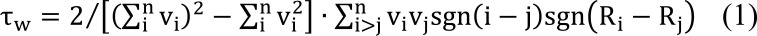


The sgn(x) = -1, 0 or 1, if x <, = or > 0. The *v_i_* represented the weighting function, which is bounded to [1, n] and ranges in (0, 1). The limiting distribution (*LD*) can be derived as Eq. 2. When *n*→∞, *LD* is approximated to *N*(0, 1), and the *p*-values can be estimated [19].


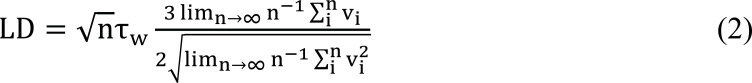


**Fig. (1):**
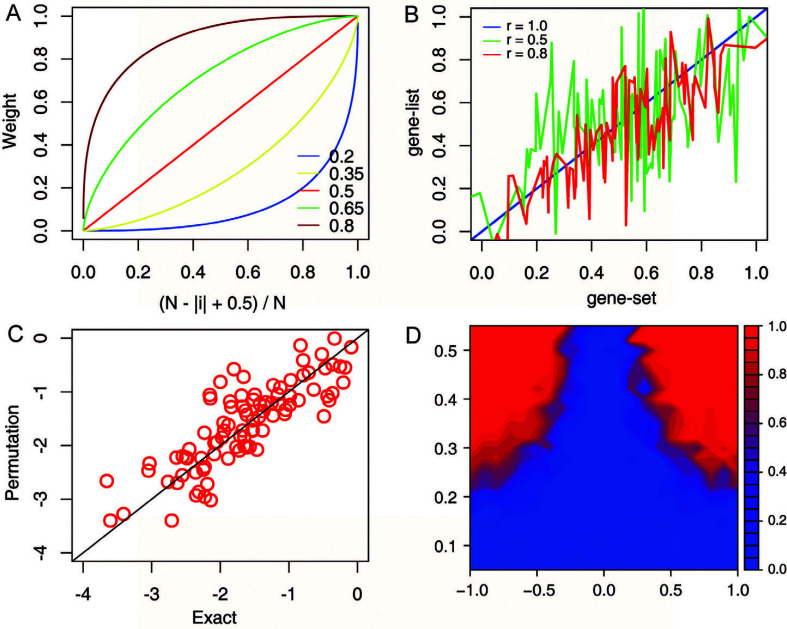
Theoretical properties of the weighted Kendall's tau rank correlation statistics. (**A**) Weighting function *v_i_*, which emphasis genes tend to have higher ranks (at 1.0) and deemphasis genes tend to have lower ranks (around 0). (**B**) Simulated testing gene sets and gene lists generated with different *r* values using the R software. (**C**) Scatter plot of the log10 transformed *p*-values estimated by Flaver's method (Exact) *versus* the corresponding log10 transformed *p*-values estimated by 10,000 times permutation approximating method. (**D**) Positive rate of the Flaver's results on testing datasets with different *q* (y-axis) and *r* (x-axis) values.

The weighting function *v_i_* used in the testing is specified Eq. 3, where *i* ranges from 1 to *n* and *p* ranges from 0.2 to 0.8 step 0.15, which consisted of a total of 5 functions (Fig. **1A**). We calculate the weight *v_i_* for each gene based on the rank *i* in gene set (-w 3), gene list (-w 2), or both (-w 1), to address specifical questions, such as see how the top-ranked genes in a gene set correlated with the gene list.


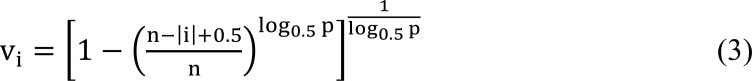


### 2.2 Development of the Flaver Software

Utilizing the weighted Kendall's tau statistics, we developed a software called “Flaver” to identify the key gene sets playing roles within a given transcriptome profile. The tool takes the ranked gene set (specified by the -s option) and the gene list (the transcriptome profile specified by the -i option) as input. Running the tool within the command window will produce a result file (-o option) containing the statistical results of the gene sets. There are five major steps built into the program: (i) match the genes between the gene sets and gene list; (ii) sort genes first by ranks of the gene sets and then by the ranks of the gene list; (iii) calculate the tau statistics for each gene set using Eq. 1; (iv) calculate the *p*-values for the significance of each gene sets using Eq. 2; and (v) perform multiple testing correction for all *p*-values using the FDR method [20]. The source code has been deposited into GitHub and is available under an academic-free license.

### 2.3 Testing Datasets for Flaver and its Competitors

Simulated testing gene sets and gene lists were generated in a fashion to represent typical data structures found in real gene expression rank data using the R software version 4.2. A normally distributed gene set data (μ = 0, σ = 1) for *i* genes consisting of a total of *j* samples was generated, named the *S_ij_*. A correlated gene list data (μ = 0, σ = 1) for *i* genes consisting of a total of *j* samples was generated, named the *L_ij_*, where sample *L_j_* was correlated with *S_j_* with a randomized correlation coefficient *r* between -0.8 and 0.8 (Fig. **1B**). Ranked gene set and gene list were created by mixing *m* random genes from the *S_ij_* or *L_ij_*, dataset with *n* random genes from a normally distributed dataset (μ = 0, σ = 1), where *q* = *n*/*m* ranges from 0.15 to 0.55 steps 0.025, respectively.

The first real-world gene set used in this study was created by the human TF to target gene interactions obtained from the Grit website release 2023 [16], named “Grit-set”. The ranks of the genes in Grit-set are the log2-transformed FDR values of Grit's output which we called Score of Binding-site (SBD). The real-world gene lists used in this study was generated based on the Protein-Atlas databases RNA consensus tissue and cell-line gene data section, version 21.1 [21], using the following processing method. Expression profile of each tissue was selected and the consensus nTPM were normalized using the quantile method.

The differential of expression (DE) is defined as DE*_i_*(g) = e*_i_*(g) – mean(e*_j-other_*(g)), which is the difference between the consensus nTPM for gene g in sample *i* and mean value of other samples (*j-other*). To evaluate the possibility that a large DE is due to chance rather than a reflection of true differential expression, we calculated *P*-values for the DE based on one-sample Student's *t*-statistics. The *P*-value for gene g in the target sample was compared across other samples to test whether gene(g) expression level was significantly higher or lower than in other samples. The *P*-values were corrected by the FDR method [20].

The results were converted into *RDE* values (Rank of Differential Expression) in the ranked gene list using Eq. 4 as recommended by GSEA's method. The tissue gene list was named “RNA55T-list”, and the cell-line gene list was named “C176-list”.


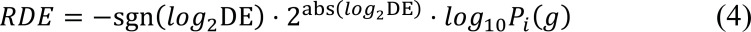


### 2.4 Performance Assessment Methods for the Flaver Software

Currently, two major classes of algorithms are implemented in gene set enrichment analyzing softwares. We focused on testing two representatives, the GSEA [12] and GOStats [13], which implement the Kolmogorov–Smirnov statistics and Hypergeometric test. To avoid biased comparison, the Gene set for GSEA and GOStats was obtained from MSigDB [12, 22] and specifically optimized for algorithms implemented in these softwares. For the RNA55T-list and C176-list, the TFs expressed in a tissue-specific manner, significantly higher or lower expressed in the target tissue than in other tissues (*P_i_*(*g*) < 10^-3^) referred to as TFs-gold, were used to benchmark the Flaver's results (*P*-value < 0.05 were referred to as RES-sig). True positives (TP) were defined as the number of overlapped TFs between TFs-gold and RES-sig. False positives (FP) were defined as the number of TFs presented in RES-sig but not in TF-gold. True negatives (TN) were defined as the number of TFs that were neither presented in RES-sig nor in TFs-gold. False negatives (FN) were defined as the number of TFs that are not present in RES-sig but presented in TFs-gold. Performance was assessed by calculating sensitivity [Sen=TP/(TP+FN)], specificity [Spe=TN/(TN+FP)], precision [Pre=TP/TP+FP], accuracy [Acc=(TP+TN)/(TP+TN+FP+FN)], error rate [Err=1-Acc], F1 score [F1=2×Sen×Pre/(Sen+Pre)], and geometry performance 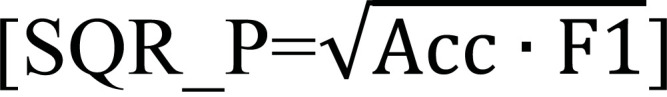 for all of the transcriptome profiles analyzed.

## 3 RESULTS

### 3.1 Theoretical Properties of the Weighted Kendall's Tau Rank Correlation Statistics

Theoretical properties of the weighted Kendall's tau rank correlation statistics were tested on a representative testing dataset that can be obtained in different situations. A matrix of different *r* (-0.8, 0.8) and *q* (0.15, 0.55) values were considered. First, the *p*-values estimated by Flaver's method and the 10,000 times permutation approximating method are highly consistent, as the correlation coefficient reaches 0.94 (*p*-value < 0.05, Fig. **1C**), indicating that Flaver's implementation of the weighted Kendall's tau rank correlation statistics was appropriate. Second, the positive rates were associated with *q*, the ratio of correlated genes; when *q* is higher than 0.25, 95% of the gene sets can be detected by Flaver (*p*-value < 0.05). An interesting observation from the results characterized by a region around *r* = 0 (not correlated) in which whatever the *q* value is, 95% of the gene sets cannot be detected (Fig. **1D**). This is reasonable because when the number of correlated genes increasing, the Flaver's statistical result tend to become more and more significant.

### 3.2 Creation of Three Ranked Gene Sets

A total of 401 PWMs for human transcription factors were obtained from the HOCOMOCO database. The promoter sequence set (1.1K-set) was obtained from the Grit’s site. The Grit run took 6 hours on a 64-core Dell R730 server. Grit identified a total of 5.27 million significant TFBS (FDR ≤ 0.05). The Grit-set was created by assigning target genes to TFs if the gene was found to be at least one TFBS for the TF. Each TF-(target gene) pair was assigned a rank value calculated by -log10, transforming the FDR of the Grit’s output for its TFBS.

A total of 828 human H3K27ac cell-line Chip-Seq datasets and 564 human H3K27ac tissued Chip-Seq datasets were collected from the Chip-Atlas database. The information on the Chip-Seq datasets can be found in Table **S1**. The Grit-set-tissue and Grit-set-cline were created by extracting the overlaps between the Grit-set and regions identified by the H3K27ac Chip-Seq technique, respectively. The rank value for each TFBS was calculated by averaging the -log10 transformed FDR calculated by Grit and the -log10 transformed MACS2 Q-value obtained from the Chip-Atlas datasets.

### 3.3 Identification of Tissue or Cell-line Specific Genes

A total of 55 human tissues and 176 human cell-line transcriptome data were collected for the Protein-Atlas database. We first identified groups of tissue or cell-line-specific genes that are dominantly or recessively expressed in a particular sample. The list of tissues and cell lines can be found in Table **S1**. To determine whether a gene is dominantly or recessively expressed in a sample, we defined DE as the difference between the observed expression level in a sample *versus* the averaged expression level across all the samples. We defined a gene as a tissue or cell line specifically expressed if it had an FC >2.0 and FDR <10-3. On average, 3,235 cell-line-specific genes and 4,412 tissue-specific genes are identified in this analysis and named as Diff_sets. As an evaluation, we performed a gene search according to the gene’s GO annotations and found an average of 23% overlap between the genes annotated with tissue-specific cell-line specific GO-terms and the Diff_sets. This means many genes with known tissue or cell-line-related functions are also dominantly or recessively expressed in the corresponding transcriptome data and vice versa. The RNA55-list and the C176-list were created by ranking the genes by the RDE values as specified by Equation 4.

### 3.4 Analyzing Gene Sets in Human Transcriptome Data Using Flaver

**Fig. (2):**
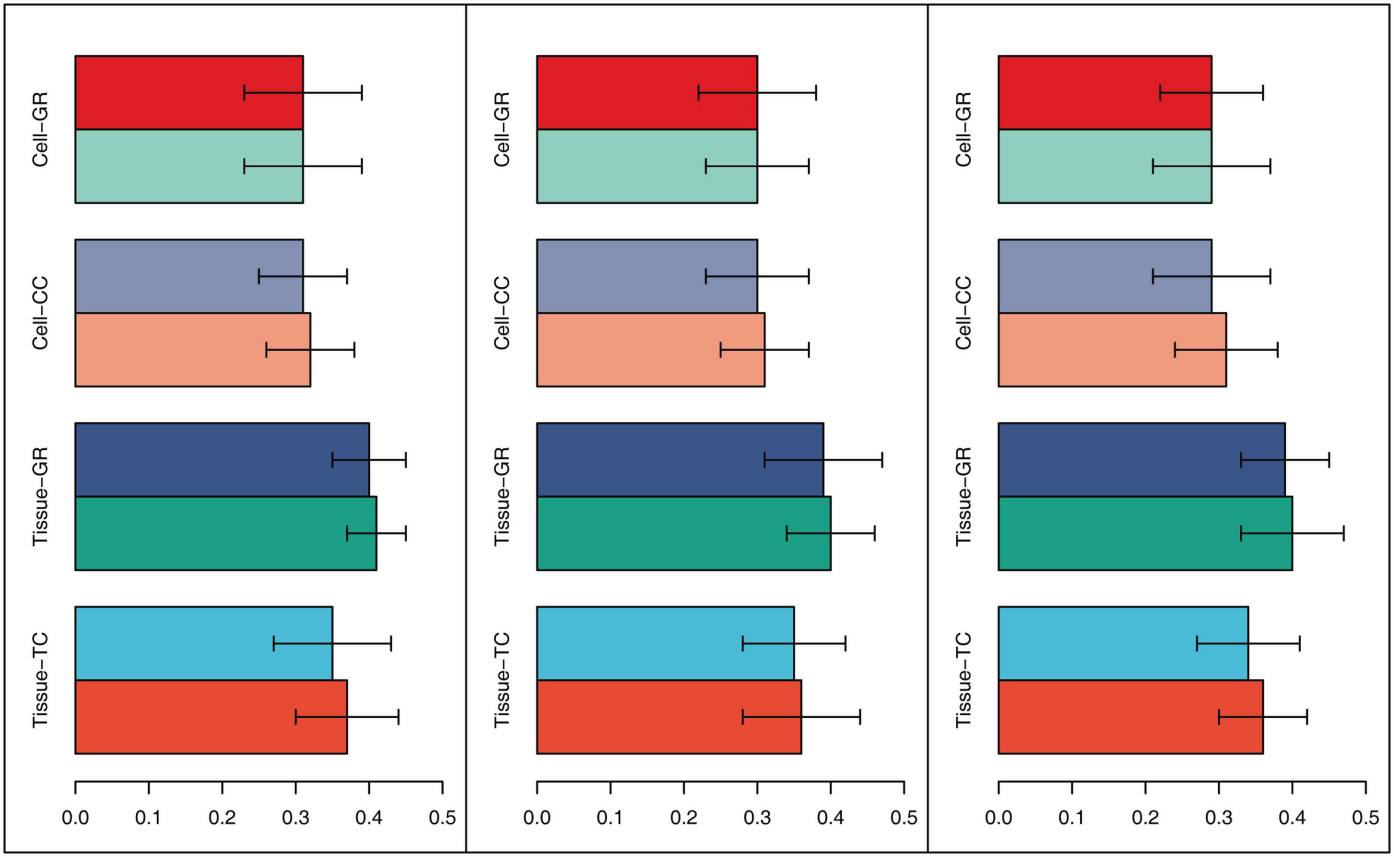
Assessments of Flaver's weighting methods. Three weighting methods, by gene set (-w 3, right panel), by gene list (-w 2, middle panel), or by both (-w 1, left panel), were evaluated based on the value of geometry performance (SQR_P). The “TC,” “CC,” and “GR” codes represent the Grit-set-tissue, Grit-set-cline, and Grit-set, respectively. The first bar in the group shows the value of SQR_P for auto *p* run (*p* ranges from 0.2 to 0.8 step 0.15, -p option on), and the second bar shows the value of SQR_P for *p* = 0.5 run.

Flaver was used to analyze the Grit-set, Grit-set-tissue, and Grit-set-cline in the human transcriptome by applying it to the RNA55T-list and C176-list. Genes in the ranked dataset and ranked gene list were run with each weighting function, and the results with the lowest FDR were retained. Although there were no significant differences among different weighting methods or between the auto *p* and the linear (*p* = 0.5) weighting function, the weighting method (-w 3) with linear weight function produced slightly higher SQR_P on average (Fig. **2**). Thus, these best parameters were set for the following analysis.

**Fig. (3):**
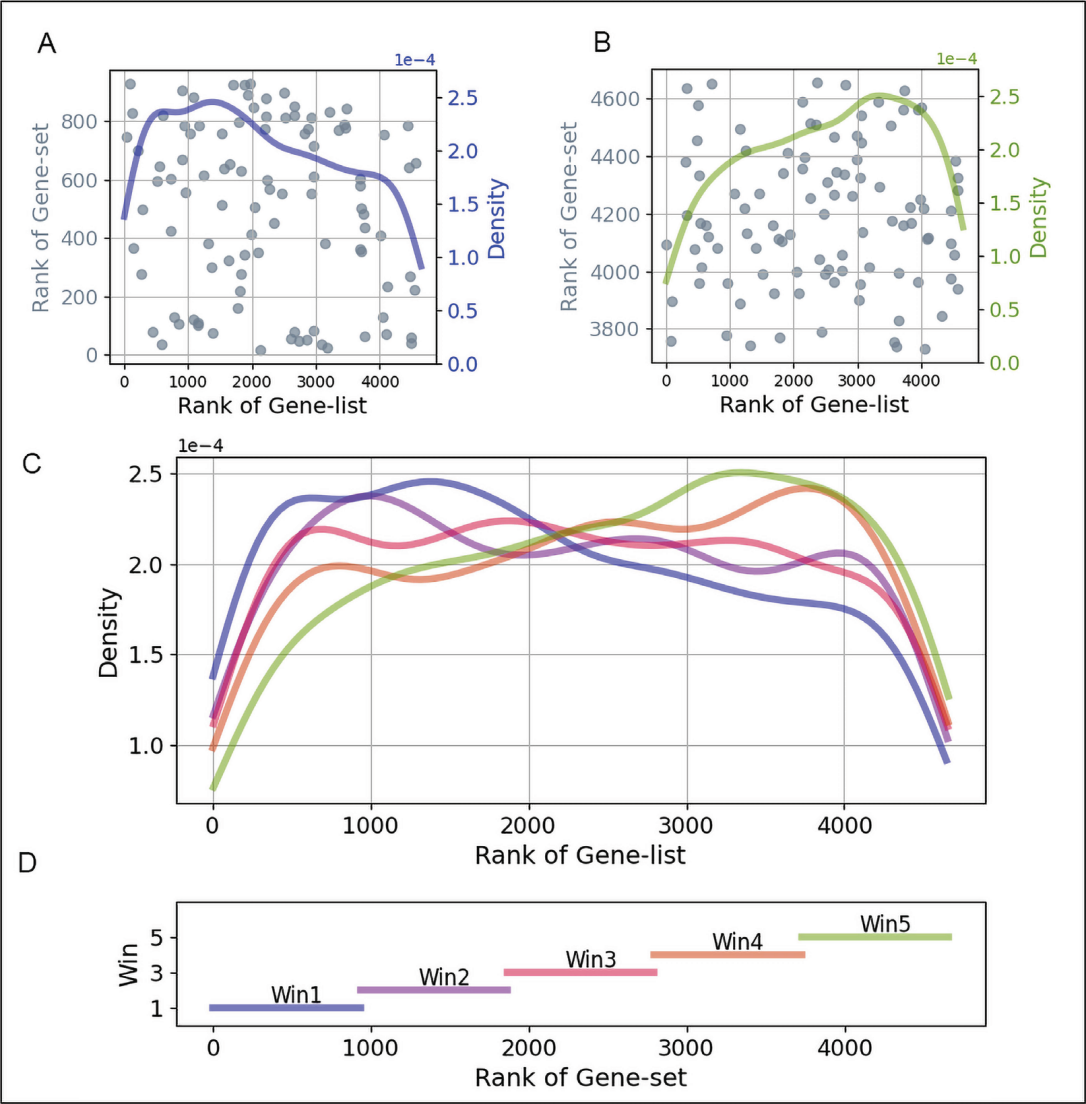
For a technical visualization of Flaver’s result, take the MEF2A gene set in the skeletal muscle gene list as an example. The distributions of the *RDE* values in sliding-window slides from the top *SBD* values to the bottom for a positively correlated example were shown in plot C. The length of the sliding window is 0.2n, and there is no overlap between each of the two windows (plot D). The top-ranked genes in the gene set also exhibit top ranks in the gene list (plot A), and the lowly ranked genes in the gene set also exhibit lower ranks in the gene list (plot B), indicating that it’s a positive correlation.

We identified a total of 2,118 Gene sets in the RNA55T-list and 4,529 Gene sets in the C176-list, of which the *SBD* values of the genes in the gene set were significantly correlated with the *RDE* values of the genes in the gene list. Tables **1** and **2** listed the top two significant gene sets per tissue sample and cell-line sample, respectively, and the full data is available in Supplemental Material **1**. For further technical validation, we visualized the distribution of the *RDE* values in sliding-window slides from the top *SBD* values to the bottom. The length of the sliding-window is 0.2*n.* There is no overlap between each of the two windows The results indicated that, for positively correlated examples, the top-ranked genes in the gene set also exhibit top ranks in the gene list, the lowly-ranked genes in the gene set also exhibit lower ranks in the gene list, where vice versa in negatively correlated examples. These illustrations indicated that our method is outstanding in detecting gene sets in which the gene ranks correlated with the genes' ranks in the transcriptome data (Figs. **3A**, **B**, **C** and **D**).

### 3.5 Differences Among Flaver and its Competitors

**Fig. (4):**
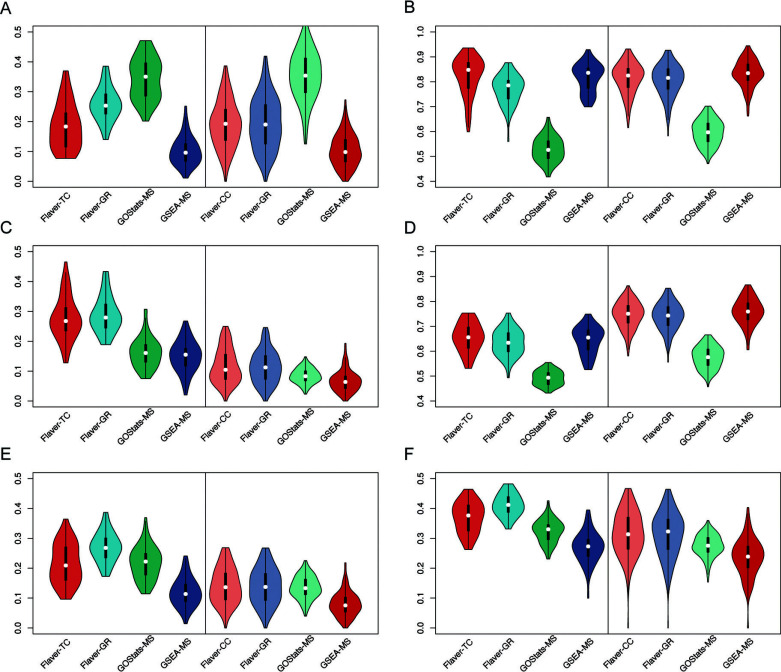
Performance assessment of the gene set analyzing tools. Three software, Flaver, GSEA, and GOStats, were evaluated based on the parameters of sensitivity (Sn, plot A), specificity (Spe, plot B), precision (Pre, plot C), and accuracy (Acc, plot D), F1 score (F1, plot E), and geometry performance (SQR_P, plot F). The “TC,” “CC,” “GR,” and “MS” codes represent the Grit-set-tissue, Grit-set-cline, Grit-set, and Gene set-MS, respectively. The tissue results were shown in the left panel, and the cell-line results were shown in the right panel for each plot. Calculations followed by sensitivity [Sen=TP/(TP+FN)], specificity [Spe=TN/(TN+FP)], precision [Pre=TP/TP+FP], accuracy [Acc=(TP+TN)/(TP+TN+FP+FN)], error rate [Err=1-Acc], F1 score [F1=2×Sen×Pre/(Sen+Pre)], and geometry performance 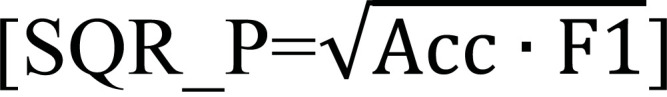 for all of the software.

The Flaver’s results were assessed with two publicly available gene set analyzing tools (Fig. **4**). The Sen of GOStats is higher than Flaver and GSEA (*p*-value < 0.05, Fig. **4A**). However, the Spe and Acc are lower than Flaver and GSEA (*p*-value < 0.05 Figs. **4B** and **D**), the Pre of Flaver is the highest among all competitors (*p*-value < 0.05, Fig. **4C**). The F1 of Flaver is higher than GSEA and GOStats for RNA55T-list (*p*-value < 0.05, Fig. **4E**). Flaver obtained the highest average SQR_P, followed by GOStats and GSEA (Fig. **4F**). The SQR_P values for Flaver were higher than GOStats (9% for RNA55T-list, p-value ≤ 0.05), indicating that Flaver performed better than GOStats for RNA55T-list. Furthermore, the Flaver method outperformed the GSEA method (14% for RNA55T-list and 17% for C176-list on average, p-value ≤ 0.05), indicating that Flaver performed better than GSEA for both RNA55T-list and C176-list. The complete assessment results for all the tools have been provided in Supplemental Material **1**.

## 4 DISCUSSION

The significant gene sets identified by Flaver may due to (i) the rank of *RDE* values of genes in the RNA55T-list globally correlated or reverse correlated with corresponding *SOD* values in Grit-set (*p* tend to be 1, example in Fig. **3**); (ii) the correlation only significant for the top rank genes in Grit-set (*p* tend to 0); (iii) or correlated based on linear weights determined by the weighting function (*p* values in the middle). It has been noticed that the number of gene sets identified by GSEA GOStates was significantly lower than Flaver, and the overlaps are less than 30%. The possible reason could be that these competitors could not use the rank information of the genes in the gene sets, but Flaver presented a solution for it.

The theory of GSEA is testing the locations of genes from a gene set within the gene list sorted by expression value *via* calculating an Enrichment Score (ES). The ES reflects the degree to which a gene set is overrepresented at the extremes (top or bottom) of the entire ranked gene list. The enrichment score corresponds to a weighted Kolmogorov–Smirnov-like statistics [12]. When the rank locations of the genes from a gene set within the gene list shift to one side, the GSEA works well. When the genes were symmetrically distributed, GSEA could not detect these gene sets. However, if the ranks of the genes in the gene set and the ranks of the genes in the gene list were significantly correlated with each other, even though symmetry is distributed, a strong association of the gene set and gene list may exist, and these gene set must be emphasized.

Typically, gene sets are several hundreds of genes in length. However, only a small part of the top-ranked items are usually informative. This small part of items is characterized by a strong correlation of their rank positions with the gene list. Identify this small part of genes to estimate the degree of agreement of the order of ranks between the gene set and gene list. Former workaround methods always set an arbitrary cutoff to the genes in the ranked gene set and used the gene above the threshold as input in the analysis. However, it is always painful to define the appropriate cutoff. Furthermore, traditional gene set enrichment analysis may not work well for the gene sets that contain both up-regulated and down-regulated genes. If the rank information of the genes in the gene set were established and this rank order is significantly correlated with the gene ranks deduced from the expression value, these gene sets must be identified. Thus, new algorithms need to be developed, which leads to the development of a new generation of gene set analyzing software.

As pointed out by Sanatgar *et al.*, in situations in which two independent sources rank n objects, interests were primarily on the agreement of the top ranks, whereas disagreements on items at the bottom of the ranks are of little or no importance [23]. One way of measuring agreement in two sets of ranks, with emphasis on the top ranks, is by computing the ordinary correlation coefficient on suitably chosen scores, and the Savage scores, which are expected values of order statistics from the exponential distribution, was a good example [24]. Then, a concordance measure is provided that is more sensitive to agreement on the top ranks, and the statistics used are functions of the ordinary correlation coefficient computed on Savage scores [25]. Their statistics for the two-sample case are shown to provide a locally powerful rank test for a model given by Hájek *et al*. [26]. A weighted Kendall's tau statistics is proposed as a more flexible solution to measure weighted correlations [19]. It can emphasize items with high ranks more than those with low ranks, or vice versa, by customizing the weighting function [19]. The idea under Flaver software is to adapt the weighted Kendall's tau statistics to transcriptome data analysis, allowing for the identification of key gene sets whose rank order is correlated with the corresponding rank orders of the genes in the gene list. The statistical inference on the key gene sets is based on correlating the ranked gene sets and ranked gene lists by an informative top-down algorithm. The significantly correlated gene sets with a high-ranked gene list detected by the algorithm are good candidates that play roles in the biological process studied.

## CONCLUSION

For gene sets identified by Flaver that are regulatory candidates to tissue function, the corresponding TFs with binding sites for genes in these gene sets should also present a tissue-specific expression profile to undertake their transcriptional regulation roles. In other words, the differentially expressed TFs among tissues can be used as a candidate golden set that can be used to benchmark the performance of Flaver and its competitors. The results indicated that the TFs identified by our method have a higher tendency to be differentially expressed across the tissues analyzed than its competitors, which showed higher Sn, Spe, Pre, and Acc. However, one should bear in mind that the abundance of a TF’s transcripts is not always consistent with the abundance of the corresponding protein, although, in most situations, they should correlate. However, it is beyond the scope of the presented study to investigate possible exceptions from this case.

## Figures and Tables

**Table 1:** Top significant TF-derived gene sets identified in human tissues.

**Tissue**	**Top2 TF-derived Gene Sets**	***P*-value*_max_***	**Tissue**	**Top2 TF-derived Gene Sets**	***P*-value*_max_***
Testis	RFX2(+); RFX1(+)	1.01E-18	Smooth-muscle	WT1(+); KLF6(+)	3.35E-05
Thymus	NRF1(+); E2F5(+)	2.69E-14	Vagina	RFX1(-); HIC1(-)	3.49E-05
Fallopian-tube	RFX2(+); HIC1(+)	1.49E-11	Gallbladder	ETV1(-); FOSB(+)	3.73E-05
Bone-marrow	ELF1(+); ELF2(+)	4.51E-11	Amygdala	ETV1(-); ELK1(-)	5.64E-05
Lymph-node	ETS1(+); ELF1(+)	6.03E-11	Midbrain	ETV1(-); FEV(-)	5.75E-05
Pituitary-gland	RFX1(+); ETV4(-)	6.45E-11	Kidney	HNF1B(+); ZNF467(-)	6.61E-05
Pons	ETV1(-); FEV(-)	3.57E-09	Spinal-cord	RFX2(+); RFX3(+)	7.49E-05
Choroid-plexus	RFX2(+); PAX6(-)	7.17E-09	Hippocampal-formation	ETV1(-); ELK1(-)	0.000109
Retina	RFX3(+); SP4(+)	8.82E-09	Small-intestine	HNF4A(+); HNF4G(+)	0.000159
Tonsil	MBD2(+); E2F1(+)	8.43E-08	Ovary	SNAI2(-); KLF15(+)	0.000176
Tongue	MEF2D(+); MYOG(+)	1.28E-07	Duodenum	HNF4A(+); HNF4G(+)	0.000185
Appendix	ETS1(+); SPI1(+)	1.32E-07	Medulla-oblongata	FEV(-); RFX2(+)	0.000351
Cerebral-cortex	ELK1(-); LHX2(+)	1.55E-07	Salivary-gland	IRF7(-); KLF5(-)	0.000539
Thalamus	ELK1(-); ETV1(-)	7.66E-07	Hypothalamus	CREM(+); ETV1(-)	0.000619
Spleen	RFX1(-); ETS1(+)	1.21E-06	Adrenal-gland	RORA(+); FEV(+)	0.000832
Skeletal-muscle	MEF2D(+); MYOG(+)	1.89E-06	Epididymis	TGIF1(+); OSR2(+)	0.000935
Parathyroid-gland	MITF(+); EHF(+)	2.69E-06	Cerebellum	ETV1(-); ZBTB18(-)	0.001055
Seminal-vesicle	WT1(+); SRF(+)	5.66E-06	Thyroid-gland	ATF3(+); MBD2(+)	0.001785
Basal-ganglia	ELK1(-); RFX3(+)	5.89E-06	Esophagus	SREBF2(-); OSR2(-)	0.001816
Heart-muscle	MEF2A(+); ESRRB(+)	6.80E-06	Endometrium	WT1(+); GLI3(+)	0.002398
Pancreas	ZNF467(-); KLF15(-)	8.89E-06	Cervix	RFX1(-); SNAI2(-)	0.002426
Liver	HNF4A(+); HNF4G(+)	1.53E-05	White-matter	FEV(-); HOXB8(+)	0.003404
Rectum	KLF4(+); KLF3(+)	1.88E-05	Breast	FOSB(+); PRRX2(-)	0.003572
Placenta	ERG(+); FLI1(+)	2.03E-05	Lung	AHR(-); NR4A1(-)	0.004249
Adipose-tissue	SNAI2(-); FOSB(+)	2.51E-05	Colon	FOXP2(-); HNF4A(+)	0.009116

**Table 2:** Top significant TF-derived gene sets identified in human cell lines.

**Cell-line**	**Top2 TF-derived Gene Sets**	***P*-value*_max_***	**Cell-line**	**Top2 TF-derived Gene Sets**	***P*-value*_max_***
HCC1937	ZFP42(-); NKX3-1(-)	6.29E-11	OCI-Ly3	SPI1(+); SPIB(+)	2.40E-05
BHY	AHR(-); ZNF121(-)	1.47E-08	697	KLF12(-); FOSL2(-)	2.41E-05
MV4-11	WT1(-); KLF1(-)	1.06E-07	OS-RC-2	ARID5B(-); JUN(+)	2.46E-05
SF268	TEAD4(+); ELK1(-)	2.48E-07	HOS	SRF(+); KLF15(+)	2.60E-05
HOP-92	ETV1(-); AHR(-)	3.56E-07	ALL-SIL	KLF12(-); KLF6(-)	2.72E-05
RD-ES	RFX2(+); GABPA(-)	4.01E-07	PF-382	USF1(-); RXRB(-)	2.75E-05
SW837	ZNF770(-); AHR(-)	8.79E-07	Toledo	E2F1(+); MECP2(+)	2.78E-05
Malme-3M	ETV1(-); LEF1(+)	1.26E-06	Rh30	MYOD1(+); E2F1(+)	2.89E-05
SK-LMS-1	SNAI2(-); WT1(+)	1.39E-06	NCI-H1963	RFX3(+); KLF12(-)	3.20E-05
NCI-H69	ETV1(-); ETS1(-)	1.89E-06	SW403	BATF(-); HNF4G(+)	3.54E-05
HT115	HNF4A(+); HNF4G(+)	3.77E-06	RPMI-8226	KLF15(-); ZNF257(-)	3.70E-05
SK-UT-1	SNAI2(-); ETS1(-)	4.10E-06	HAP1	KLF15(+); E2F5(+)	3.76E-05
HT	ETS1(+); ELF1(+)	6.55E-06	NCI-H446	E2F7(+); ETV1(-)	3.88E-05
MOLM-13	WT1(-); IKZF1(-)	6.69E-06	Karpas-299	HOXB4(-); GFI1(-)	4.04E-05
Karpas-422	JUND(-); ELF1(+)	8.53E-06	JH-EsoAd1	MYCN(-); TFAP2A(+)	4.38E-05
A2780	SNAI2(-); ZEB1(-)	9.27E-06	Loucy	FOSL2(-); RXRB(-)	4.72E-05
HCC1599	ELK4(-); AHR(-)	1.10E-05	RL	CREM(-); ETS1(+)	4.76E-05
U2OS	ZNF467(+); TEAD4(+)	1.27E-05	HCC827	JUN(+); ETS1(-)	4.97E-05
MUTZ-3	ZNF121(-); ERG(+)	1.84E-05	NB4	WT1(-); IKZF1(-)	5.24E-05
NCI-H2887	E2F5(-); HOXA13(-)	1.92E-05	SW620	WT1(-); IRF7(-)	5.25E-05
SNB-75	ETV1(-); EGR2(+)	1.95E-05	RD	ETV1(-); TEAD4(+)	5.67E-05
HOP-62	WT1(+); ETV1(-)	2.03E-05	MKN1	ELF3(-); FOS(+)	5.85E-05
SU-DHL-1	ATF3(-); ARID5B(-)	2.04E-05	MEC-1	ETS1(+); MEF2B(-)	6.28E-05
TT	ETV1(-); HOXB4(-)	2.26E-05	SET-2	MEF2C(-); GFI1(-)	6.31E-05
MDA-MB-468	MECP2(-); FOXQ1(-)	2.28E-05	NCI-H520	TCF7L2(+); HOXC9(+)	6.73E-05

## Data Availability

A software implementing the method, called “Flaver,” was developed and is publicly available at http://www.thua45.cn/flaver under an academic free license. The full data presented in the manuscript is available at http://www.thua45.cn/flaver/Supplemental-Data-**1**.zip.

## LIST OF ABBREVIATIONS

DE: Differential of Expression
ES: Enrichment Score
SBD: Score of Binding-site
TFs: Transcription Factors
