# Supplementary material for: Testing the Significance of Ranked Gene Sets in Genome-wide Transcriptome Profiling Data Using Weighted Rank Correlation Statistics
Source: Curr Genomics. 2024 Mar 11;25(3):202–11. doi: 10.2174/0113892029280470240306044159 (PMC11288161; doi:10.2174/0113892029280470240306044159)
Supplement: Supplementary file 1 [file CG-25-202_SD1.zip › CG-25-202_SD1/5b-BMS-CG-2023-150 Supplementary Material.pdf]

Supplementary Material

Testing the Significance of Ranked Gene Sets in Genome-wide Transcriptome Profiling Data Using Weighted Rank Correlation Statistics

Min Yao[1], Hao He[1], Binyu Wang[1], Xinmiao Huang[1], Sunli Zheng[1], Jianwu Wang[2], Xuejun Gao[1,#] and Tinghua Huang[1,*]

[1]*College of Animal Science, Yangtze University, Jingzhou, Hubei 434025, China;* [2]*College of Agriculture, Yangtze University, Jingzhou, Hubei 434025, China*

http://www.thua45.cn/flaver/Supplemental-Data-1.zip

Supplemental-Data-1 Directory Root

---celline-tissue-flaver-assesment.xlsx Assessment Results

---flaver-cell-line Flaver Cell-line Directory Root

------analysis_result Assessment of the Flaver Result

---------analysis_flaver_res-grit-all-cell-line.py Analysis Flaver Result (Grit-set, C176-list)

---------analysis_flaver_res-grit-chipall-cell-line.py Analysis Flaver Result (Grit-set-cline, C176-list)

---------celline-flaver-chipall-w1-assesment.txt Assessment Flaver Result (Grit-set, C176-list)

---------celline-flaver-gritall-w1-assesment.txt Assessment Flaver Result (Grit-set-cline, C176-list)

------grit-human-v100-hocom-2-s2.bed Grit-set

------grit_inchip_cell_bed_merged.bedGrit-set-cline

------output-w1-e0-hocom-s2_cell_grit-all_py-norm-pp.5 Flaver Output (Grit-set, C176-list)

------output-w1-e0-hocom-s2_cell_grit-inchip_py-norm-pp.5 Flaver Output (Grit-set-cline, C176-list)

------run_flaver-cell-grit-all-w1.py Run Flaver (Grit-set, C176-list)

------run_flaver-cell-grit-inchip-w1.pyRun Flaver (Grit-set-cline, C176-list)

---flaver-tissueFlaver Tissue Directory Root

------analysis_reault Assessment of the Flaver Result

---------analysis_flaver_res-grit-all-tissue.py Analysis Flaver Result (Grit-set, RNA55-list)

---------analysis_flaver_res-grit-inchip-tissue.py Analysis Flaver Result (Grit-set-tissue, RNA55-list)

---------tissue-chipall-flaver-w1-assesment.txt Assessment Flaver Result (Grit-set, RNA55-list)

---------tissue-gritall-flaver-w1-assesment.txt Assessment Flaver Result (Grit-set-tissue, RNA55-list)

------grit-human-v100-hocom-2-s2.bed Grit-set

------grit_inchip_tissue_bed_merged.bed Grit-set-tissue

------output-w1-e0-hocom-s2_tissue_grit-all_py-norm-pp.5 Flaver Output (Grit-set, RNA55-list)

------output-w1-e0-hocom-s2_tissue_grit-inchip_py-norm-pp.5 Flaver Output (Grit-set-tissue, C176-RNA55-list)

------run_flaver-tissue-grit-all-w1.py Run Flaver (Grit-set, RNA55-list)

------run_flaver-tissue-grit-inchip-w1.py Run Flaver (Grit-set-tissue, RNA55-list)

---gene-list-cell-prep-py-norm C176-list

---gene-list-cell-prep-py-norm-e5 C176-list-E-5

---gene-list-tissue-prep-py-norm RNA55-list

---gene-list-tissue-prep-py-norm-e5 RNA55-list-E-5

--- gostats GOStats Directory Root

------analysis_gostats_res-cell-norm-gseatft-l5.py Analysis GOStats (MSigDB, C176-list)

------analysis_gostats_res-tissue-norm-gseatft-l5.py Analysis GOStats (MSigDB, RNA55-list)

------c3.tft.v2023.1.Hs.symbols.gmt.txt MSigDB-tft

------c3.tft.v2023.1.Hs.symbols_dataframe.txt MSigDB-tft-dataframe

------cell-gostats-gseatft-assessment.txt Assessment GOStats Results (MSigDB, C176-list)

------celline-2-gsea-tft GOStats Output (MSigDB, C176-list)

------GOstats-11-08.R Run GOStats

------tissue-2-gsea-tft GOStats Output (MSigDB, RNA55-list)

------tissue-gostats-gseatft-assessment.txt Assessment GOStats Results (MSigDB, RNA55-list)

------universe-celline.txt Full Gene-List

------universe-tissue.txt Full Gene-List

---GSEA_4.3.2 GSEA Directory Root

------analysis_gsea_celline_tft_py_l5_p.05.py Analysis GSEA (MSigDB, C176-list)

------analysis_gsea_tissue_tft_py_l5_p.05.py Analysis GSEA (MSigDB, RNA55-list)

------c3.tft.v2023.1.Hs.symbols.gmt MSigDB-tft

------celline_gsea_tft_assesment.txt Assessment GSEA Results (MSigDB, C176-list)

------celline_gsea_tft_out_dit.txt GSEA Output Dir list

------gene-list-cell-prep-py-norm C176-list

------gene-list-cell-prep-py-norm-e5 C176-list-E-5

------gene-list-tissue-prep-py-norm RNA55-list

------gene-list-tissue-prep-py-norm-e5RNA55-list-E-5

------GSEA_4.3.2 GSEA Directory Root

---------out_gsea_win_GSEA_tft_celline GSEA Output (MSigDB, C176-list)

---------out_gsea_win_GSEA_tft_tissue GSEA Output (MSigDB, RNA55-list)

---------run_gsea_win_GSEA_tft_celline-py-norm.py Run GSEA (MSigDB, C176-list)

---------run_gsea_win_GSEA_tft_tissue-py-norm.py Run GSEA (MSigDB, RNA55-list)

------tissue_gsea_tft_assesment.txt Assessment GSEA Results (MSigDB, RNA55-list)

------tissue_gsea_tft_out_dir.txt GSEA Output Dir list
